# Water and Sewerage of Cities

**Published:** 1869-09

**Authors:** 


### WATER AND SEWERAGE OF CITIES.

One of the most remarkable histories of modern times, showing the extraordinary, prompt and decided influence which a proper drainage has on the health of cities, is found in General Butler's government in New Orleans. For several years in succession, immediately previous to his taking up his headquarters there, it had been desolated with malignant types of various diseases, terminating in the most fatal forms of yellow fever. It was regarded as a fixed fact by the Confederacy that "Yellow Jack" would make quick work, and sweep off the Northern army to a man; but not a single case of yellow fever occurred the first season, nor the second, nor, in fact, any epidemic disease, simply because intelligent minds kept the city clean by a judicious system of drainage, and the Northern army suffered no more from sickness than would have occurred in ordinary circumstances.

More than forty years ago, Louisville, Ky., was the hot-bed of malignant diseases that were at times terrible. Educated medical men, in lectures, in personal conversation, and through the newspapers pointed out the connection between the epidemics and ponds of standing water covered with a noisome green scum in various parts of the city during the summer months.

Finally the authorities were awakened on the subject, every pond was either cleaned or drained off or filled up, and the most astonishingly prompt change for the better in the health of the people took place, and from that day Louisville has not only been one of the very healthiest cities in the great Mississippi Valley, but has quadrupled its wealth and population, and now promises, in its uninterrupted prosperity, to be one of the most prominent cities of our country, while its colleges and schools of learning are the resort of both sexes from the most distant parts of the South and West.

A dozen years ago, Rahway, N. J., was annually afflicted with various forms of fevers and other diseases. So palpably did this arise from stagnant mill-ponds, that finally, impatient with long suffering and the "red tape" of the authorities, the people took the matter in hand and tore down the mill-dams, with the result that it became at once a delightful and healthy place of abode, with constant accessions of new and valuable citizens ever since.

We observe in one of our oldest and best exchanges, the *Poughkeepsie Eagle*, a paper noted for the variety and excellence of its columns, that a public meeting of the citizens of that delightful burgh has been held, and that measures are about to be taken to make Poughkeepsie one of the very healthiest, as it is one of the most favored, localities on the noble Hudson for wealth, culture and enterprise. This has the more attracted our attention from its being the home of the Vassar College for young ladies, four hundred of whom, our daughters and sisters, crowd its already famed portals from every portion of the Union. Not only this, but the schools for the youth of both sexes in Poughkeepsie have been noted for their superior excellence for upwards of a quarter of a century, and still maintain their ancient fame; but it occurs to us that it is not merely the duty and interest of the people to do all that is practicable to increase its healthfulness and its desirableness as a residence, but a common humanity dictates that no ordinary expense should be spared in making the city as well-known for its salubrity and general healthfulness. Let Poughkeepsie get rid of its shallow, stagnant ponds, and quagmires, pay the owners a fair compensation, if the riddance would not be a benefit to them as well as to the city at large, and make a running stream of the Fallkill; let a city, boasting of its twenty thousand inhabitants and "a heaven-kissing hill," with a broad river at its base, be at least as well supplied with pure, wholesome water, and as well sewered as nature has provided means at hand. And then Poughkeepsie, with all its splendid advantages of location, would be the "Mecca" of families of intelligence and wealth, retiring from the bustle and temptations of our city life, either from motives of personal health and comfort, or for the better education and bringing up of their children, provided once and always that they could "Croton-ize" their dwellings from cellar to attic.

We are aware that within the past few years filtering cisterns have become a necessity and a make-shift with many of its inhabitants, but the great mass of the people still use the the water of hard limestone wells, with not a foot of sewerage, but abundance of cess-pools in the town, and carry the water by hand through their houses as their wants require.

What have the people of Poughkeepsie been thinking about in this nineteenth century? We say that they owe it to their intelligence, their honor, and their humanity, to push forward at this late day the improvements indicated without the delay of another hour. They owe it to their intelligence, for in not doing it they are far, very far behind the spirit of the age; to their honor, for how can they otherwise invite the youth of the country to come there for an education while in the pursuit of which they must take their chances of undermining their constitutions by miasmatic influences; to their humanity, in that pure air, abundant water and means of cleanliness are due to that great mass of every community, the laboring classes, under whose energies all cities flourish and to whom so much of the comfort and happiness of all are indebted.

The importance of wholesome water and good sewerage to every single dwelling cannot be overestimated, and any city neglecting this vital matter must expect to suffer at all times, and particularly when an epidemic of any kind sweeps over the country.

Poughkeepsie will remember the cholera of 1849, twenty years ago, when, commencing with the death of one of its own physicians (Dr. Slater), there were many fatal cases of that terrible disease among its citizens.

Standard medical works abound with information on this subject, but the general intelligence of the people freely appreciates the value of cleanliness, abundant water, and efficient sewerage in promoting the health and happiness of all communities.

